# The American Agriculturist

**Published:** 1876-01

**Authors:** 


*The American Agriculturist* holds fast to its old name, which it has carried successfully through the long period of *thirty-four years,* and commences the "Centennial year" with the vigor of the prime of life, and with well founded promises of still greater achievements in its appropriate sphere—that of a plain, practical highly instructive and trustworthy family journal. Its name is almost a mis-nomer because it is now equally useful to city, village and farm.

The closing number of volume 34, now before us, like its usual issues, is full of good things, varied in contents, which are prepared with much labor, thought and care, and illustrated with over 60 well executed and well printed original sketches and engravings.—This *Journal* is a marvel of cheapness, beauty and utility, costing only $1.60 a year, postage included, for its more than 500 double pages of useful information, and 500 to 600, or more, of fine engravings. It will interest everyone. Orange Judd Company, Publishers, 245 Broadway, New York city.

